# A conversation with Evan Weber, PhD, assistant professor, The Children’s Hospital of Philadelphia/University of Pennsylvania

**DOI:** 10.1017/cts.2025.10166

**Published:** 2025-10-03

**Affiliations:** Clinical Research Forum, Washington, DC, USA

## Top 10 Clinical Research Achievement Awards Q & A

This article is part of a series of interviews with recipients of Clinical Research Forum’s 2025 Top 10 Clinical Research Achievement Awards. This interview is with Evan Weber, PhD, Assistant Professor, The Children’s Hospital of Philadelphia/University of Pennsylvania. Dr Weber’s research focuses on developing approaches to enhance therapies for pediatric cancer by reprogramming T cells with improved durability and exhaustion resistance. He received a 2025 Top 10 Clinical Research Achievement Award for “Memory Reprogramming Enhances CAR T Cell Efficacy.” *The interview has been edited for length and clarity.*

## When did you first become interested in clinical research?

Ever since I was very young, maybe 10 or 12 years old, I’ve been interested in science and specifically the life sciences. I spent most of my adolescence and early adulthood thinking that the only outlet for that interest was to be a physician—probably because I didn’t know any scientists. I followed the pre-med track in college, and it wasn’t until I was starting a gap year before med school that I had any real experience in a research setting. I got a job in a neuroscience lab and to be honest, it was awful, really horrible. My responsibilities included running cognitive behavioral experiments with rats, and it was a very odd experience that kind of turned me off.

## Oh, no! What changed your mind?

Luckily, that was just a short stint. When it ended, I was serendipitously connected to a PI who needed someone to help him move his lab. I started out as the lab manager and eventually started to take on more scientific responsibilities, working with clinical fellows, postdocs, PhD students, and other technicians. What struck me was that they all were constantly learning—without relying on textbooks—and that really connected with my learning style. In many ways, the research we were doing felt like play, testing interesting ideas and using cool machines to study cells at a molecular level.

## Is this when you decided to pursue a PhD?

Yes. Even though I had already taken the MCAT and applied and interviewed at med schools, I ended up deciding to pursue a PhD instead. I’ve always been a people person, and when I was younger, I guess I never imagined that academia would be the right fit for me. In some corners it might not be, but what I’ve come to realize is that academic research, when it’s done right, is based on relationships, collaborations, public presentations, and communication, all of which involve working with people.

## How did your early research experiences lead to the award-winning paper about enhancing Chimeric Antigen Receptor (CAR) T cell efficacy?

I made a big transition from PhD to postdoc. For my PhD, I worked in a basic science immunology lab, studying how white blood cells infiltrate inflamed tissue. That’s a very specific process that’s necessary for any inflammatory response, but it has nothing to do with cancer or engineering T cells. At the time, I was keeping an eye on the field of cancer therapeutics, and I can still remember the moment in 2012 when I first read about CAR T cell therapy in *The New York Times.* The article highlighted work by Dr Carl June, who’s a pioneer in immunotherapy and now my colleague at the University of Pennsylvania, and I was so inspired that when it was time to look for a postdoc, I quite naively emailed the biggest names in the field. One of those who responded was Dr Crystal Mackall at Stanford, and she became my mentor and is the co-senior author on the paper that was selected for this award.

## What aspect of CAR T cell efficacy did you study in this paper?

As a postdoc, I explored a lot of different concepts around making CAR T cells work better at fighting cancer. One of the major limitations of CAR T cell therapies is the poor persistence of these cells in patients, and we know that the expression of memory-associated genes is linked to their long-term persistence and the most durable clinical responses. For this paper, we investigated two T cell transcription factors to see if they are responsible for promoting T cell memory. We tested TCF1, which we thought would likely be important, and in parallel we interrogated an unorthodox factor called FOXO1.

## What did the results show?

We showed that overexpression of FOXO1 promotes memory and restrains exhaustion in human CAR T cells. That means overexpressing FOXO1 can increase the antitumor activity of human CAR T cells, and it suggests memory reprogramming can be a broadly applicable approach for optimizing therapeutic T cell states. Overexpression of TCF1 did not have the same effect.

## You mentioned that this paper was a collaborative effort. What type of collaborations were involved?

First, this was an effort between multiple labs: my lab, Dr Mackall’s lab, and the lab of Dr Ansuman Satpathy, who worked with us on the computational analysis. The collaborative effort between our three labs was amazing, including during the publication process, which can be laborious and stressful. In addition, I want to point out another level of collaboration. We co-submitted our manuscript alongside two groups from the Peter MacCallum Cancer Centre in Australia, led by Drs. Paul Beavis and Phil Darcy.

## How did these collaborations benefit the research?

Science is moving in a more interdisciplinary direction, especially in the CAR T cell space which merges concepts of immunology, bioengineering, cancer research, pharmacology, and more. That means collaborations are becoming increasingly crucial. But beyond that, when we learned that there were other labs working on something very similar, with results that complemented ours, it turned out to be a major inflection point—not just for this project, but for my career. It was the first time I’d encountered this, and we had to decide if we wanted to compete or join forces. Our groups had never met, and they were literally halfway across the world, but we started with a simple conversation around our projects, and that eventually led to an incredible partnership. Making that connection just sort of cracked the whole problem open and accelerated all of our efforts. We compared data and talked through our findings to make sure there wasn’t too much overlap and then coordinated our submissions. Ultimately, it was a hugely transformative experience because it showed that there doesn’t have to be this zero-sum game competition. Instead, everyone can win and that can, in turn, advance the field even faster.

## How do you stay motivated?

What has always motivated me about this job has just been doing fun science and working with great people. If what we’re doing is creative and can make an impact, and as long as I have people around me who are energetic and curious, it’s not hard to do this job. Actually, it’s a privilege.

